# Supplementary material for: A Link between Handgrip Strength and Executive Functioning: A Cross-Sectional Study in Older Adults with Mild Cognitive Impairment and Healthy Controls
Source: Healthcare (Basel). 2022 Jan 26;10(2):230. doi: 10.3390/healthcare10020230 (PMC8872145; doi:10.3390/healthcare10020230)
Supplement: Supplementary file 1 [file healthcare-10-00230-s001.zip › healthcare-1512902-supplementary.pdf]

Table S1. Overview of the general characteristics of the participants.

General characteristics of the participants	Median ± Interquartile Range (Minimum to Maximum)		
	**aMCI (*n* = 22; 14f/ 8m)**	**naMCI (*n* = 21; 9f/ 12m)**	**HC (*n* = 27; 19f/ 8m)**
**aHGS left / right [in kg]**	26.3 ± 18.3 / 29.8 ± 14.4 (19.8 to 49.7 / 20.3 to 50.0)	31.4 ± 13.4 / 34.5 ± 20.4 (17.9 to 51.6 / 19.6 to 54.6)	26.7 ± 10.6 / 28.9 ± 13.4 (10.0 to 40.1 / 14.1 to 42.9)
**MMSE [z-score]**	−1.88 ± 0.93 (−3.31 to 0.37)	−1.87 ± 1.21 (−2.94 to 0.44)	−0.77 ± 0.74 (−2.08 to 0.64)
**TMT A [in seconds]**	44.5 ± 15.8 (26.0 to 79.0)	41.0 ± 20.0 (28.0 to 101.0)	38.0 ± 18.0 (24.0 to 69.0)
**TMT A [z-score]**	−0.24 ± 1.30 (−1.92 to 2.43)	−0.09 ± 1.31 (−2.32 to 1.63)	0.36 ± 1.28 (−1.24 to 2.62)
**TMT B [in seconds]**	109.0 ± 34.3 (51.0 to 227.0)	115.0 ± 60.0 (67.0 to 195.0)	85.0 ± 57.0 (51.0 to 184.0)
**TMT B [z-score]**	−0.46 ± 1.32 (−2.30 to 3.07)	−0.24 ± 0.92 (−2.00 to 1.32)	0.55 ± 1.99 (−1.52 to 1.86)
**TMT B/A**	2.25 ± 1.07 (1.34 to 5.28)	2.73 ± 0.92 (1.45 to 3.90)	2.40 ± 0.81 (1.43 to 5.17)

aHGS: absolute handgrip strength; MMSE: Minimal Mental State Examination; TMT: Trail Making Test. Please note that values of aHGS left in the naMCI group were based on $n = 20$ since the data of one participant in the naMCI was not used to calculate median, interquartile range, and minimum to maximum due to drop hand symptomatic in the left (non-dominant) hand.
